# Left Atrial Thrombus Presenting with Acute Coronary Syndrome and Cerebrovascular Event

**Published:** 2018-04

**Authors:** Ahmet Güner

**Affiliations:** *Department of Cardiology, University of Health Sciences, Division of Kosuyolu Heart & Research Hospital, Istanbul, Turkey.*

**Keywords:** *Heart atria*, *Thrombosis*, *Acute coronary syndrome*, *Myocardial infarction*, *Stroke*

A 50-year-old female patient was admitted to the emergency department with a cerebrovascular event and acute anteroseptal myocardial infarction. Computed tomography showed acute focal ischemia at the right frontal lobe. She was transferred to the catheterization laboratory. Diagnostic coronary angiography via the right femoral artery route revealed normal right and left circumflex coronary arteries and an image suggestive of a thrombus in the distal left anterior descending artery (Figure 1). Subsequently, the left main ostium was engaged with a 7F Judkins-4 guiding catheter. A 0.014-inch floppy guide wire was advanced distally in the left anterior descending artery. After several attempts to perform thrombus aspiration, a thrombolysis in myocardial infarction (TIMI) 3 flow was obtained. Control transthoracic echocardiography demonstrated apex-wall hypokinesis and a large mobile left atrial mass, 42×15 mm in size (Figure 2). The mass was freely mobile in different planes and was free floating. It was prolapsing into the mitral valve as far as the tips of the mitral valve leaflets from the left atrium, but it was not prolapsing across the mitral valve. In the foreground, the transthoracic echocardiographic findings were consistent with an atrial myxoma. For further evaluation, transesophageal echocardiography was performed, which showed the presence of a nonhomogeneous mass, 42×15 mm in size, filling the left atrial appendix and the presence of a partially free-floating mass, 40×20 mm in size, in the left atrium but attached to the main mass by a thin bridge (Figure 3 and Figure 4). The patient successfully underwent surgery, and the primary operative finding was a spherical purple gelatinous mass (Figure 5). Histopathological examination confirmed a layered, partly necrotic mostly organized thrombus (Figure 6).

Left atrial masses mainly consist of tumors, vegetations, and thrombi. Despite the availability of many imaging modalities, it may still be difficult to differentiate a left atrial thrombus from a myxoma. Surgery is, however, the best way to confirm the diagnosis.

**Figure 1:**
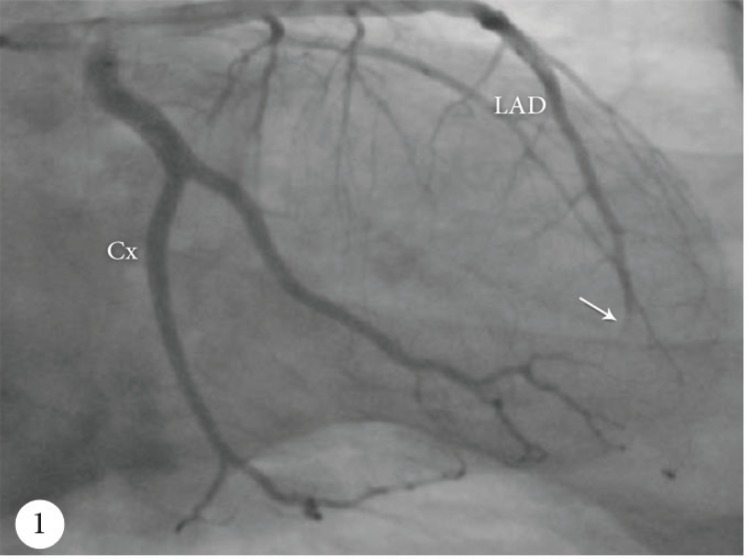
Right caudal angiographic view, showing the distal occlusion of the left anterior descending artery by a thrombus (arrow).

**Figure 2:**
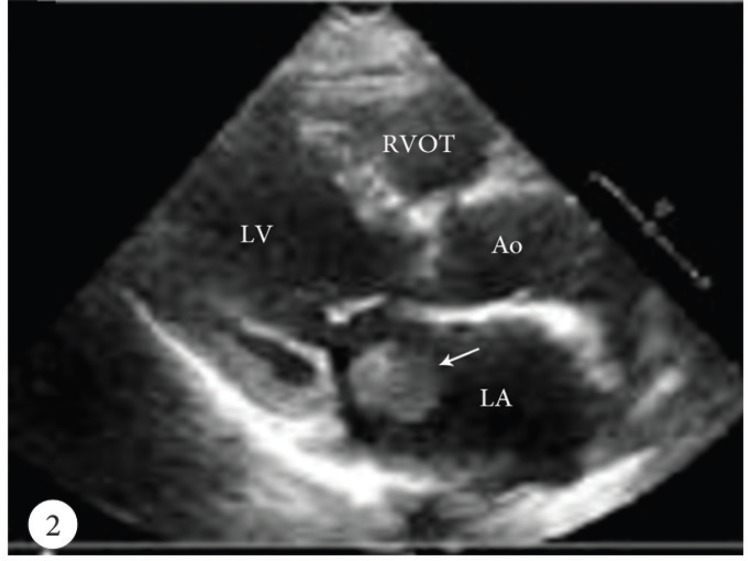
Parasternal long-axis view of transthoracic echocardiography, showing a non-homogenous spherical mass in the left atrium, simulating a myxoma (arrow).

**Figure 3:**
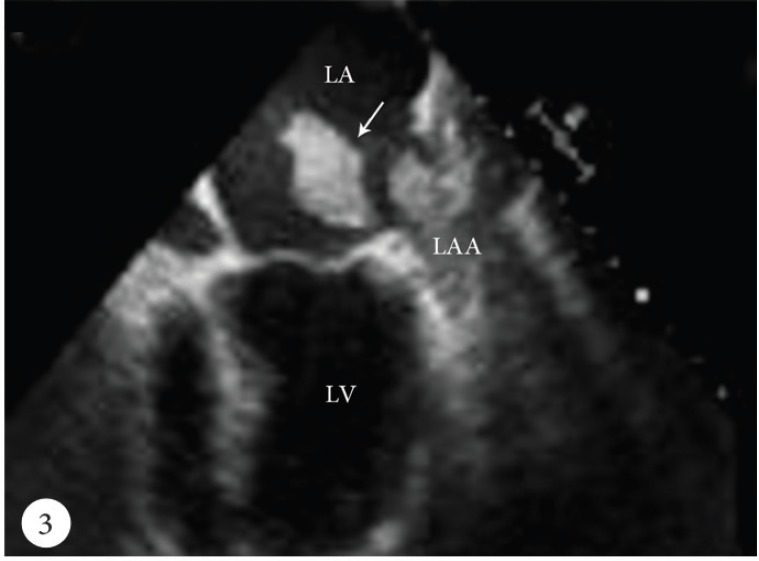
Mid-esophageal 2-chamber view of transesophageal echocardiography, showing a round mass in the left atrium and a big mass filling the left atrial appendix (arrow).

Ao, Aorta; Cx, Circumflex artery; LA, Left atrium; LAA, Left atrial appendix; LAD: Left anterior descending artery; LV, Left ventricle; RVOT, Right ventricle outflow tract

**Figure 4:**
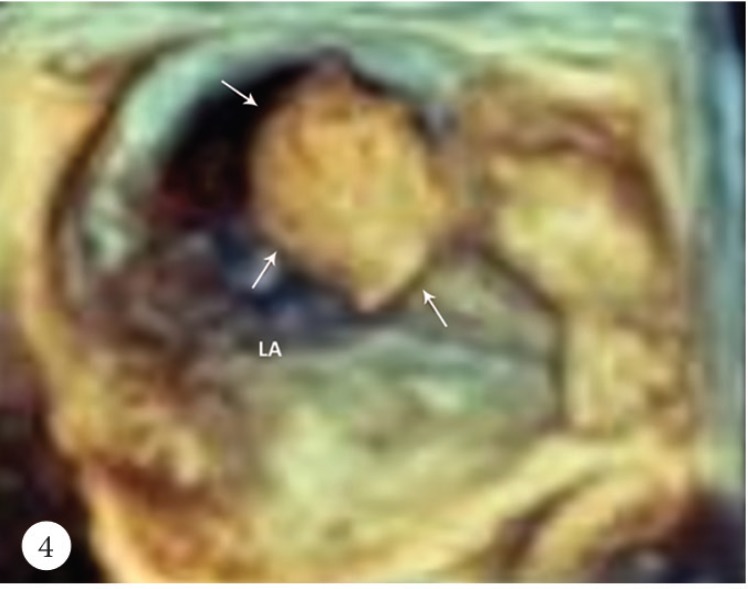
Three-dimensional transesophageal echocardiography, showing a huge thrombus filling the left atrial appendix and a highly mobile part attached to the main mass by a thin bridge (arrows).

**Figure 5:**
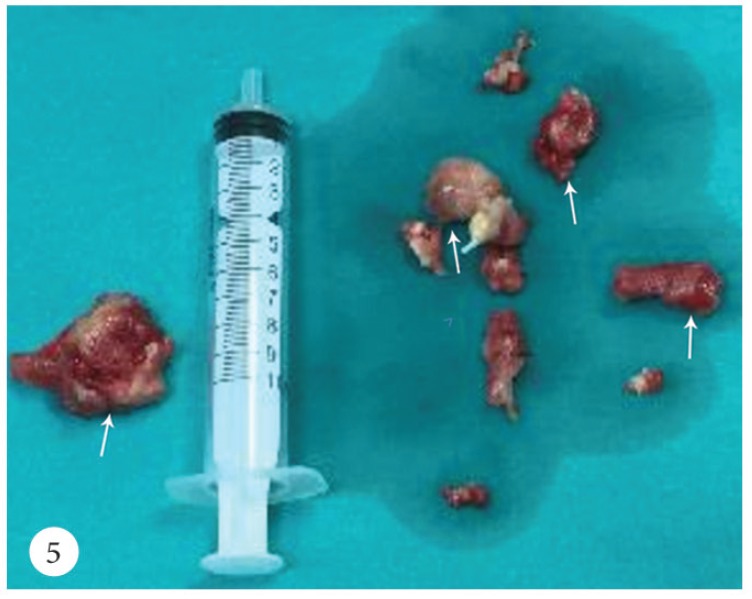
Macroscopic image of the left atrial thrombus (arrows)

**Figure 6:**
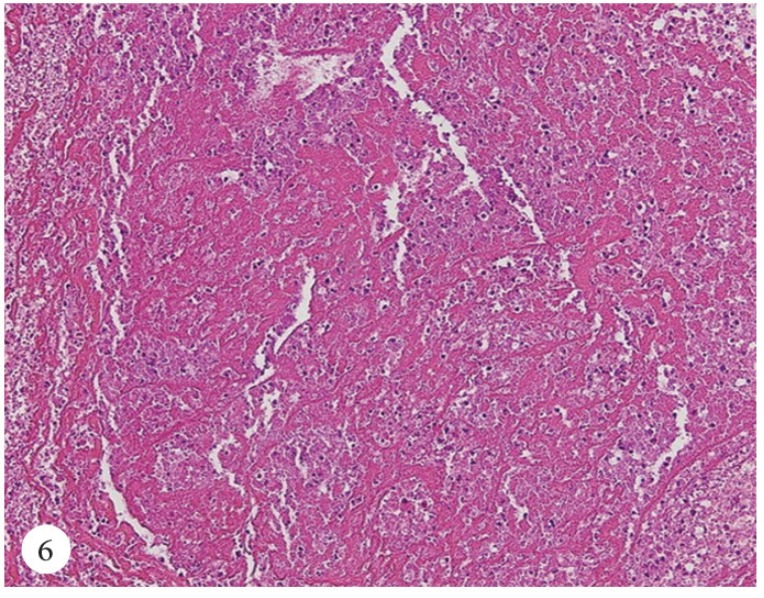
Histopathological image of the left atrial thrombus, which consists of red blood cells and fibrinous materials

LA, Left atrium

## Notes:

***This paper should be cited as: ***Guner A. Left Atrial Thrombus Presenting with Acute Coronary Syndrome and Cerebrovascular Event. J Teh Univ Heart Ctr 2018;13(2):99-100.

